# Discussion of off-target and tentative genomic findings may sometimes be necessary to allow evaluation of their clinical significance

**DOI:** 10.1136/jme-2023-109108

**Published:** 2023-06-20

**Authors:** Rachel H Horton, William L Macken, Robert D S Pitceathly, Anneke M Lucassen

**Affiliations:** 1 Clinical Ethics, Law and Society, Wellcome Trust Centre for Human Genetics, Oxford, UK; 2 Centre for Personalised Medicine, St Anne's College, Oxford, UK; 3 Clinical Ethics, Law and Society, University of Southampton, Southampton, UK; 4 Department of Neuromuscular Diseases, UCL Queen Square Institute of Neurology, London, UK; 5 NHS Highly Specialised Service for Rare Mitochondrial Disorders, Queen Square Centre for Neuromuscular Diseases, The National Hospital for Neurology and Neurosurgery, London, UK

**Keywords:** ethics- medical, genetic counseling, databases- genetic, informed consent, ethics

## Abstract

We discuss a case where clinical genomic investigation of muscle weakness unexpectedly found a genetic variant that might (or might not) predispose to kidney cancer. We argue that despite its off-target and uncertain nature, this variant should be discussed with the man who had the test, not because it is medical information, but because this discussion would allow the further clinical evaluation that might lead it to becoming so. We argue that while prominent ethical debates around genomics often take ‘results’ as a starting point and ask questions as to whether to look for and how to react to them, the construction of genomic results is fraught with ethical complexity, although often couched as a primarily technical problem. We highlight the need for greater focus on, and appreciation of, the ethical work undertaken daily by scientists and clinicians working in genomic medicine and discuss how public conversations around genomics need to adapt to prepare future patients for potentially uncertain and unexpected outcomes from clinical genomic tests.

## Introduction

The National Health Service (NHS) aspires to be the first national healthcare system to offer genome sequencing as part of routine care.^1^ Genome sequencing catalogues a person’s entire genetic code, aiming to find the 4.1–5 million points (variations) at which their DNA differs from the ‘standard’ or ‘reference’ sequence.^2^ These variations are then sifted depending on the questions being asked. In a clinical context, this is typically to try to find an explanation for medical symptoms that are thought to be genetic in origin.

The initial filtering of a person’s genomic variations aiming to find a genetic diagnosis for existing health problems is automated—pipelines developed by clinicians and scientists aim to ‘shortlist’ variants for further review. The automated shortlisting will involve discarding variants, for example, because they are common in the general population (on the premise that they are unlikely to cause severe genetic conditions or more people would be affected). Virtual panels may also be applied, where shortlisted variants are restricted to only those within a prespecified list of genes, chosen because of their potential relevance to the symptoms a person is experiencing.^3^ ^1^ Variants that make it through automated filtering processes are then scrutinised by scientists and/or clinicians charged with deciding whether the variants may have relevance to the person’s medical history.^4^

In addition to trying to explain a person’s current medical presentation, it is possible to ask further questions of their genomic data, ranging from where their recent ancestors might have lived, to their susceptibility to COVID-19.^5^ The capacity of genomic testing to do this is well appreciated and often ‘additional findings’ are offered to people having genomic tests. For example, the American College of Medical Genetics and Genomics recommends that 73 ‘medically actionable’ genes, unrelated to the reason for testing, should be analysed for people having exome or genome sequencing.^6^ The 100 000 Genomes Project in the UK offers participants analysis of 13 genes where certain variants may increase their risk of developing various health conditions.^7^ Such plans position ‘additional information’ from the genetic code as being of value, but also suggest that they are something over which we should have control—‘additional findings’ are something we might choose to seek out, rather than being thrown up unexpectedly in the course of genomic analysis.

Political discourses around genomic testing often position it as giving clarity and certainty—for example, the policy paper *Genome UK: the future of healthcare* discusses how ‘Genomics is revolutionising the way we think about healthcare. It is providing us with a far more detailed understanding of what causes illness and infectious disease and is underpinning the development of new interventions that would have been unthinkable even a decade ago’.^8^ Perhaps because of such discourses, many people expect genomic testing to be informative.^9^ However, the knowledge base regarding the medical impact of genomic variations is continuing to shift and in some cases be overturned as more is learnt about the wide range of benign variation.^10^ Relatively often in the course of genomic testing, variants are identified that may have an impact on health, but the evidence regarding this is conflicting or relatively weak. These ‘variants of uncertain significance’ pose a challenge as they have potential to be misunderstood both by clinicians^11^ and patients.^12^

Here, we discuss a case, recently discussed at the UK Genethics club, which fuses two challenges of clinical genomic testing: (A) the capacity to generate information unrelated to the reason for testing and (B) the difficulty of determining the clinical significance of genomic variation. In this case, genomic testing identified a variant of uncertain significance that could not account for the muscle weakness that prompted the patient to have the test, but that might (or might not) predispose to kidney cancer. We argue that this finding does not yet constitute a clinical ‘result’, but that disclosure is warranted in order to make possible the gathering of more information that might allow it to become so.

## Case

A man develops muscle weakness and has multiple clinical tests which are unable to establish an explanation or cause. He has genome sequencing and his data are analysed to search for a diagnosis. As part of the analysis, a ‘virtual’ panel of genes linked with muscle weakness is applied to the man’s genomic data (aiming to find variants that he has within these genes). The panel includes a gene called fumarate hydratase (*FH*), as if both of a person’s two copies of the *FH* gene are not working properly it causes a severe syndrome (fumarate hydratase deficiency) which includes muscle weakness.

This analysis finds a variant in just one copy of the man’s *FH* gene that has not previously been recorded on any publicly available genetic database (ie, as far as we can tell it has not been seen by a diagnostic or research laboratory before). This would not explain his muscle symptoms. However, certain variants in one copy of the *FH* gene predispose to a form of kidney cancer that is often aggressive and tends to be diagnosed at a late stage. People with such variants also tend to develop skin leiomyomata (benign lumps in the skin that can be very subtle and therefore not come to medical attention) and in women, uterine fibroids (non-cancerous growths in the womb that sometimes cause symptoms like heavy menstruation). In families with this sort of variant in *FH*, individuals are offered yearly MRI scans of their kidneys from late childhood onwards, because around 15% will develop kidney cancer and treatment is more likely to be successful where cancer is detected early. *FH* is not on the list of genes offered as ‘additional findings’ by the 100 000 Genomes Project, or recommended for opportunistic analysis by the ACMG,^6 7^ that is, it is not a gene where there is currently a precedent for seeking out cancer-predisposing variants in the absence of an indicative personal or family history (in contrast to for example, the *BRCA* genes which predispose to breast, ovarian, prostate and pancreatic cancer), and was only analysed here because of its link to muscle weakness.

Based on current scientific evidence, it is not known whether the man’s particular *FH* variant falls into the category that predisposes to kidney cancer since its effects are hard to predict. If the man’s *FH* variant really does increase his risk of kidney cancer, this is likely to be somewhat lower than 15% over his lifetime. This is because our understanding of the risk conferred by *FH* variants comes from studying families who have been tested and diagnosed because multiple members have had kidney cancers—and even in these families, the majority of people with a cancer-predisposing *FH* variant will not develop kidney cancer. In people without this sort of family history, it may be that there are other genetic and unknown factors at play that make the *FH* variant less likely to lead to kidney cancer.^13^ For this man, the risks are even vaguer as his *FH* variant is not definitively associated with kidney cancer.

The man is not known to have skin lumps or a family history of uterine fibroids or kidney cancer, but a person with muscle weakness would not routinely be asked about these, so it is possible that he does, unknown to his clinicians. It would be possible to ask him this information in retrospect, but this would likely require some explanation as to why these questions are now being asked.

## Does the *FH* variant constitute medical information?

We consider the arguments for and against communicating a possible risk of kidney cancer as an outcome from a genomic test intended to explain muscle weakness. We argue that the genomic variant identified by the test should not yet be considered a clinical ‘result’, but has the potential to transform into one (or to fade into insignificance) if more information were gathered. Disclosure is warranted in order to allow gathering of this information, aiming to clarify the significance of the variant and advance understandings of genomic variation for future patients.

### If an optimal filtering system would not have shown this variant, can we ignore it?

Genomic testing was offered to this man in the hope that it could explain his muscle weakness. This finding does not do that. Does that have a bearing as to whether we should consider it a ‘result’ of his test? As previously discussed, the ability for genomic tests to find health-relevant information beyond the reason for testing is well known, and the willingness of initiatives to offer such information is predicated on a belief that there is value in such information. If the man had been asked if he did or did not want ‘additional information’ from genomic testing, his answer might provide a steer as to whether he might regard the *FH* variant as useful information, though empirical research indicates that many patients would expect to be told if unexpected medically relevant information is found in the course of genomic testing, even if it lies outside what they have provided specific consent to,^9 14 15^ and that most people say ‘yes’ if asked at the outset whether they would want additional information from genomic testing. For example, in the 100 000 Genomes Project, most people opted to receive ‘additional findings’.^16^

However, the *FH* variant found in this man is both off-target (it does not explain his muscle weakness) and uncertain (its link to kidney cancer is theoretical, not established). The automated software used to filter the man’s genomic data was designed to shortlist this variant for human scrutiny only because if the patient’s symptoms closely matched fumarate hydratase deficiency syndrome, further analysis of the *FH* gene might be warranted to search for a second variant in pursuit of a diagnosis. That was not the case here—the patient’s muscle symptoms were very different to those experienced by people with fumarate hydratase deficiency, so having reflected on and rejected that possibility, would it not be acceptable for this variant to be discarded as irrelevant to the purpose of the test?

Another issue that this case highlights is the challenge of choosing which genes to include on a panel. Fumarate hydratase deficiency (the condition resulting when both of a person’s copies of the *FH* gene are not working properly) was never going to be the cause of this man’s muscle weakness—this condition affects people in infancy or early childhood, with many other symptoms in addition to muscle weakness. Yet selecting exactly which genes to analyse on an individual patient basis would be prohibitively time-consuming and hence would compromise the ability of laboratories to offer genomic testing at scale. In designing gene panels, people therefore have to navigate a tension between missing diagnoses, and generating off-target findings. However, if a bespoke virtual gene panel had been designed for this patient, *FH* would not have been included—a theoretical decision to ignore *FH* variants would have been built into the analysis from the offset, without this necessarily being recognised as having an ethical aspect.

A further point of interest in this case is that the responsibility to consider whether this *FH* variant constitutes medical information arose both because the automated filtering pipeline happened to include a gene that, while linked to related symptoms, was not going to be the cause of this patient’s health problems, but also because the person analysing the data happened to have an awareness of cancer predisposition syndromes and recognised its potential significance. If the data had been reviewed by a health professional who purely specialised in muscular disorders, and discarded a single *FH* variant because they knew it could not be the cause of the patient’s muscle weakness, without being aware that it might have different medical consequences, would we see that as a problem?

### How does the uncertainty around the *FH* variant influence its status as medical information?

One argument for considering the *FH* variant as being medical information that should be discussed with the patient, is that doing so might have the potential to prevent harm. If the *FH* variant really does predispose to kidney cancer, then alerting the patient of this risk will be important.

The counter to this is the not easily quantifiable probability that the man’s *FH* variant is benign variation, with no impact on kidney cancer risk at all. Treating the *FH* variant as medical information, and discussing it with the patient, might lead to understandable worries that he and/or his relatives might develop kidney cancer that may turn out to have no scientific foundation. These concerns may be much easier to create than to take away. However, information is not withheld in medical practice on the basis of its potential to distress—instead, thought is put into how best to communicate difficult news sensitively but accurately.

The potential to cause psychological distress could therefore be regarded as an unconvincing reason not to discuss the *FH* variant, though given the tentative and uncertain nature of this finding it is debatable to what extent it is appropriate to consider it ‘information’. The downstream implications also need consideration. For example, considering the *FH* variant as medical information might lead to initiation of annual kidney MRIs for this patient, with associated costs for the health service in providing and interpreting the scans. If it is considered appropriate to offer kidney scans to this patient purely on the basis of having a poorly understood *FH* variant, it would be only fair to offer such scans to anyone in his family who also has the variant. Conceptualising this variant as a medical result could feasibly lead to numerous scans and clinical encounters on the basis of a chance finding that may have no clinical impact, and will use up resources from a very underfunded health service.^17^

Of course, it would be possible to discuss the *FH* variant with the patient, and for the NHS to decide not to offer kidney scans based on current evidence. However, Raz takes the view that one of the conditions of autonomy is ‘an adequate range of options’, and here, if NHS screening may not be available, it is not clear what these might be.^18^ Private kidney screening might be available if the man has the means and inclination to pursue this, but if he does not, treating the *FH* variant as a medical result may lead to him being trapped with information he might prefer not to have, without enabling him to act differently.

One could argue that the *FH* variant is ‘his’ information because found in ‘his’ DNA, but the same will be true of over a million other variants, some of which will be comparably concerning based on hypothetical evidence. It is unsurprising that the man has a genetic variant that looks concerning—a 2015 analysis of 1000 Genomes participants found they had on average 24–30 variants implicated in rare disease in the ClinVar database,^2^ so we would expect that this patient might have a similar number of potentially concerning variants in his genome. The aspect that is unusual in this case is that someone else has happened to see exactly where in his genome one of these potentially concerning variants happens to be. If the medical implications of the variant were clear, serious and easily mitigated, this might create a duty of easy rescue. But that is not the case here—the variant might be entirely benign, and the method of harm prevention (regular kidney MRIs) is imperfect and burdensome.

### Implications for practice

We argue for tentative discussion of the *FH* variant with the patient, not because it constitutes medical information, but in order to allow the further appraisal that might allow it to become so. Here, skin features and a detailed family history might allow some degree of reassurance, or provide clear evidence that this variant should be regarded as medically important.

With increasing use of genomic tests in healthcare, this sort of dilemma will become more common: variants that might have potential to impact on health will be spotted by chance not by design. This is challenging in a context where deterministic expectations around genetic tests are common. For each person, at the level of an individual gene, worrying variants are very rare. Looking globally across a person’s genome, it is perfectly normal to have several variants that look, in theory, concerning. The unusual situation here is that genomic testing has created a potential opportunity for the man to know which gene one of those variants is in in him, not that he has a concerning variant in the first place. However in the context of popular discourses around genomic testing that tend to present it as clear and informative,^9^ people may be primed to hear such variants as being more medically significant than the evidence suggests.

Scientists and clinicians frequently need to strike a balance between not giving undue credence to findings that may turn out to be nothing, and missing opportunities to prevent (serious) harm. Guidance is plentiful both relating to appraisal of the technical evidence underlying variants,^4^ and regarding ‘additional findings’,^6^ yet it cannot cover every challenging situation that people practising genomic medicine are faced with. Some difficulties arise because of concerns around the gap between popular expectations of genetics, and current scientific abilities to interpret the genetic code—leading to worries that people (and their clinicians) might run ahead of the evidence in reacting to variants found. However, while on an individual level this may favour not discussing uncertain variants, on a societal level it makes no progress with the wider problem of potentially overoptimistic expectations of clarity and predictiveness from genetic testing, and in a sense perpetuates these by trying to make genomic testing conform to them, despite the uneasy fit.

Many genetic variants can be considered ‘knowledge-in-waiting’^19^—sharing and discussion are part of the process by which they may become meaningful, or fade into insignificance, but deciding at what stage wider discussion is merited is difficult. As genomic testing is increasingly offered outside of specialist settings, this case demonstrates the importance of mainstream clinicians having access to support from specialist health professionals in genomic medicine when interpreting and responding to the findings of such tests.

The case outlined here was discussed at the Genethics forum (a UK-based forum for health professionals and other interested parties to discuss and explore difficult ethical and/or legal issues encountered in genetic medicine—www.genethicsuk.org). The consensus was that in situations like these there are there no ‘off the shelf’ solutions and each case will need careful, context-attentive consideration of the pros and cons of considering an uncertain variant as potential medical information. Interestingly, no-one asked whether the patient had consented to additional findings when agreeing to have genomic testing. Clearly we cannot assume that this means attendees considered this question unimportant, but it does point to the inadequacy of consent as a basis for making all subsequent decisions relating to genomic testing.^20 21^ With the capacity of genomic testing to create difficult-to-anticipate situations, however granular the consent process there may yet be unanswered questions, and it is important to recognise the ethical challenge this presents for scientists and clinicians charged with creating ‘results’ from a person’s genetic code.

## Conclusions

We describe a case where clinical genomic testing initiated to explain muscle weakness instead finds a variant that might (or might not) increase risk of kidney cancer. We argue that this variant should be discussed with the patient, not because it constitutes medical information, but because doing so would make possible further clinical appraisal that might allow it to become so. The case illustrates the need for scientists, clinicians and society to become comfortable in the ‘messy zone’ of genomic data, and the importance of working to develop societal perceptions of genomic testing in line with technical realities, rather than suppressing data that do not conform. However, it is interesting to reflect on the responsibilities created by human oversight of genomic data—if the automated analytic pipeline had only included genes that were a plausible cause of this particular patient’s symptoms, the variant we discuss here would never have been seen, and if the person analysing the data exclusively focused on muscular disorders its potential significance might not have been noticed. Ethical debates around genomics often focus on whether to look for, or how to react to, genomic ‘results’, but the process by which variants are appraised and given the status of results also deserves attention—the challenges of developing pipelines and interpreting variants may appear purely technical, but the impacts of analytical decisions are profound and the ethical work that this creates for scientists and clinicians deserves appreciation and discussion.

## Data Availability

Data sharing not applicable as no datasets generated and/or analysed for this study.

## Footnotes

A ‘virtual panel’ aims to give results similar to a targeted gene panel. In a targeted gene panel, sequencing data are only generated for particular genes. However, a virtual panel can be applied to sequencing data from a whole exome or genome (meaning a lot more genomic data are sequenced than are analysed). Various different virtual panels can be applied to the same genome sequence, whereas targeted panels would require additional physical experiments to sequence the genes for each new panel.
